# Periods of Stress and Their Dental Marks

**Published:** 1901-10

**Authors:** James G. Kiernan


### PERIODS OF STRESS AND THEIR DENTAL MARKS.[1]

BY JAMES G. KIERNAN, M.D.[2]

DESPITE embryologic teachings, the old notion that man alone is an entity who undergoes development still controls pathology and physiology. The human being, however, is a compound animal, in whom organs have their own nervous system and their own life under control of the cerebral spinal system. Vertebrate embryos are of common type at their origin, and assume successively many common forms before definitely differentiating. The higher vertebrates contain in essence the organs and possibilities of all lower vertebrates. The human organism is, therefore, a balance. While the balance is maintained the organs work in unity, though there is a constant struggle for existence between them. During embryonic existence this struggle is more intense and diversified than after maturity because of the influence of three contending forces,—remote atavism, or retrogression to primitive types; immediate atavism, or retrogression to less remote ancestors of the same type; and, finally, immediate heredity. Remote atavism tends to preserve structures which occur in the normal embryo only to disappear. The human heart passes through all vertebrate heart phases. This is likewise true of the teeth. During the life of the embryo the tooth system wavers at one time between the polyphyodont and the diphyodont. At this period, should the diphyodont tendency of man be arrested, the polyphyodont takes its place and the human being sheds teeth as do reptiles. On the other hand, should immediate atavism gain the ascendency over remote atavism, as shown in polyphyodontia, diphyodontia occurs. When the struggle for existence between the two is keenest, a period of stress results which affects the organism as a whole. This stress expresses itself most strongly in dental and maxillary irregularities, since the jaws and teeth are among the most variable structures in evolution. Under what is known as the law of economy of growth is governed the relation of the organs to each other and the process whereby one structure is sacrificed for the development of another or for the

---

[1] Abstract of a paper read before the Section on Stomatology, American Medical Association, June, 1901.

[2] Fellow of the Chicago Academy of Medicine; Foreign Associate Member French Medico-Psychological Association.

development of the organism as a whole. Since certain parts in the evolution of organs disappear, and in the evolution of organisms certain organs through suppressive economy, and since the disappearing and developing tendency of necessity centres around the time when certain functions are to be lost by the disappearing, and others gained by the developing, periods of stress occur around which the law of economy of growth centres the struggle for existence between parts of organs and between organs themselves. It is because of this that physiologic atrophies and hypertrophies and their reverse occur. Nearly all conditions of physiologic disturbance may result at these periods of stress from the influence of maternal nutrition or environment or of hereditary factors. The fœtus, therefore, must be prepared to pass through not only intra-uterine periods of stress whose dental mark has already been indicated, but post-uterine periods as well.

The child has not attained its full development at the time of birth. It has within it certain potentialities, some of which are never fully realized. Man is an imperfectly developed child. There is a constant struggle between the central nervous system and certain bodily functions for preservation of the individual and some for preservation of the race, through which the central nervous system fails to reach the height indicated in the child. This struggle for existence after birth is keenest at certain periods. Each period is marked by dental phenomena. The first is the period of the first dentition. Here, coincident with teething, the child is gaining its impressions of the outside world, is learning to walk and talk, and is also developing its eliminative organs, especially the rectum. These varied functions constitute a strain on the system the effects of which are most often evinced through the teeth and jaws. The conditions charged to teething are an expression of constitutional strain finding its outlet through the point of least resistance. During this first dentition the strain of development forces attention to the teeth and thereby leads to neglect of other factors. The teeth at this period should be regarded as a meter of constitutional strain and not a cause of it. Within the next period, between two and six, occurs the first great check to continued development of the brain. Man has learned to use his brain despite this check, but had it not occurred he would have had a higher type of brain to use. Sometimes during this period the brain gains in size, albeit not in balance, at the expense of the general system. In no small degree the struggle for existence during this period of stress centres around the development and eruption of the sixth-year molar. With the eruption of this molar, premature puberty, sexual precocity, epilepsy, insanity, gout, rheumatism, obesity, and other nutritive degeneracies may occur. All have been charged to the eruption of the sixth-year molar, whereas its irregular or difficult eruption is like them, an expression of constitutional stress. Hygiene of the teeth at this period means also constitutional, mental, and moral hygiene. Epilepsy, for example, is not a disease, but a symptom of weakness of certain vasomotor inhibitions. The first convulsion does not constitute epilepsy. Through a law of the nervous system, nerve action, once roused, tends to repeat itself. In this way are established normal and abnormal habits, of which last epilepsy is one. In its early stages a habit, normal or abnormal, is easily checked. The first convulsion, therefore, could be prevented were its premonitions known. A recurrence could also be prevented were the constitutional origin recognized. Observation of the general constitution at this time because of irregular eruption of the sixth-year molar would enable the physician to nip epilepsy and many allied conditions in the bud. Reflex notions, however, must be flung overboard. All irritations should be removed and any constitutional irregularity treated.

The obese child of six years, even though the obesity be not excessive, should be looked upon from a health stand-point with suspicion. There is liability to disease and marked tendency to systemic weakness when under morbid influence. These children are particularly liable to rheumatism, gout, etc., and profuse hemorrhage from slight causes. Youthful obesity is sometimes associated with precocious maturity and resultant early senescence. More often it coexists with extended infantilism, as in the case of Dickens's "fat boy."

E. S. Talbot, examining two hundred and sixty-seven corpulent school-children and adolescents, found marked stigmata of degeneracy. Ninety-two per cent. had deformed ears to a marked degree. Sixty-six per cent. had arrested development, as compared with their age, while twelve per cent. presented excessive development. Thirty-four were too young to determine the form and size of the jaw. In thirty-three and a half per cent. of the thirty-four the molars, incisors, cuspids, and bicuspids were present. Ninety-six per cent. of these had small teeth. Eighty-seven per cent. (of the two hundred and thirty-three) had arrested development of the upper jaw. Twenty-two per cent. arrest of lower jaw. Sixty-four per cent. had V-shaped or saddle-shaped arches or their modification and protruding teeth. Seventeen per cent. had hypertrophy of the alveolar process. Eighty-three per cent. had small teeth. Twenty-seven per cent. had extra tubercles upon the molars. Eighty-two per cent. had stenosis of the nasal cavity more or less marked. Thirty-six per cent. had deflection of the nasal septum to the left and twenty-nine per cent. to the right. Twenty-one per cent. wore glasses for eye defect. In fifty-eight per cent. there w s enlargement of the thyroid gland and in seven per cent. arrest of its development. In two hundred and ninety-six cases of early lipomatosis (one hundred and eighty males and one hundred and sixteen females) coming under my own observation, there were ten cryptorchids, six hypospadiacs, and three cases of pseudo-hermaphroditism. Three females had infantile bifid uteri. Four had enlarged clitorides; in one of these the urethra perforated the clitoris, as in the female shrew. Of forty girls who had reached eighteen, only three had menstruated normally. The others were amenorrhœic or dysmenorrhœic, or had neurotic storms during the period. There were one hundred and sixty hebephreniacs among the number. Of these, one hundred and twenty had masturbated excessively. Ten had been nymphomaniac or satyriasic, the sexual appetite having become completely extinct at eighteen. Fifty of the non-hebephreniacs never showed any signs of sexual appetite. Three of the hebephreniacs were sexual inverts, while eighty practised various perversities. Of the non-hebephreniacs ten were cyclothymiacs, thirty had had acute forms of insanity, ten were epileptics, and fifteen hysterics. Thirteen had had chorea. Ninety-seven had difficulty in learning to speak and thirty always stuttered.

The appearance of the first permanent molar is of great significance to stomatologists and sociologists alike. The mental and moral deficiencies usually present will interfere with proper care of the teeth, particularly indicated in cases where such deficiencies occur. While the eye has received much attention as a sociologic guide, the teeth and jaws have, as a rule, been neglected, although of even more significance than the functional disorders of the eye.

The next important period of stress is practically that of the second dentition. Here there is a foreshadowing of the mental and nervous phenomena of the following period of stress. Sexual development now frequently receives its initiative. What is true of neuropathic children earlier is true at this time of ordinary children under conditions of stress. There is irregularity of or disturbed sleep, irritability, apprehension, strange ideas, great sensitiveness to external impressions, high temperature, delirium, or convulsions from slight causes, disagreeably anxious dreams, romancing, intense feeling, periodic headache, muscular twitching, capricious appetite, and marked intolerance of stimulants and narcotics. The struggle for existence between the developing alimentary and other systems from the second to the sixth year has produced effects which are most felt during the sixth to the twelfth year.

- Neuroses . . .
  - Convulsions.
  - Nervous laughs.
  - Nervous coughs.
  - Hiccoughs.
  - Stuttering.
  - "Tics."
  - Neuralgias.
  - Neurasthenia.
  - Ecstasy.
  - Hysteria.
  - Chorea.
  - Epilepsy.
  - Somnambulism.
    - Renal.
    - Hepatic.
    - Gastric.
    - Vesical.
    - Genital.
    - Pulmonary.
    - Adenopathic.
    - Cardiac.
    - Metabolic.
- Psychic Types .
  - Hallucinations.
  - Anomalies of character.
  - Aberrant sentiments.
    - Love.
    - Jealousy.
    - Anger.
  - Obsessions or imperative concepts.
    - Pure.
    - Attended by impulsive acts.
      - Arson.
      - Suicide.
      - Homicide.
      - Alcoholism.
      - Theft.
      - Rape.
      - Non - criminal acts.
  - Night terrors.
  - Idiocy.
  - Imbecility.
  - Mania.
  - Acute confusional insanity.
  - Melancholia.
  - Transitory frenzy.
  - Stuporous insanity.
  - Stupor.
  - Katatonia.
  - Paranoia.
  - Cyclothymia.

These are apt to occur at this time as a reaction to temporary disturbances of health. The temperature at which psychic disturbances begin in a child is a fair index of its brain stability. Neuropathic children on a very slight rise in temperature or even without it are subject to attacks of unreasonable elevation, during which they are quite beside themselves, rushing about, wildly shouting, fighting, and without clear consciousness of their surroundings. With this last picture stomatologists are very familiar in children brought to them for care of the teeth. During this period such disturbances have to be taken into account in cases where maxillary and dental treatment and regulation are indicated.

The period of puberty should include the period from the appearance of the menses or of spermatozoa until the completion of the twenty-fifth year. The appearance of the wisdom-tooth at this time marks human maturity, which was the reason that twenty-five was selected for the age of maturity by the Code of Napoleon. During this period it is possible to correct dental and maxillary irregularities with benefit to the subject. Every one of the conditions enumerated as due to strain during the period between six and twelve may appear. In addition there occurs a type of mental disorder called hebephrania, or insanity, of puberty, which is practically incurable after its development. This is an insanity peculiarly charged to cigarette-smoking, masturbation, over-study, religious excitement, and "love," all of which are the expressions of the defects and not their cause. These subjects in the earlier stage of their psychosis peculiarly require teeth regulation. In some cases the strain from the tooth irregularity may have sufficed to upset the unstable mental balance. The next great periods of stress in human life are those of the climacteric and of the senile period. The first is accompanied by the menopause in women and prostate change in men. The dental conditions marking these two periods of stress are generally involutional in character and require prosthesis, or treatment, not correction.

